# Global DNA Adenine Methylation in *Caenorhabditis elegans* after Multigenerational Exposure to Silver Nanoparticles and Silver Nitrate

**DOI:** 10.3390/ijms24076168

**Published:** 2023-03-24

**Authors:** Anye Wamucho, Jason Unrine, John May, Olga Tsyusko

**Affiliations:** 1 Department of Plant and Soil Sciences, University of Kentucky, Lexington, KY 40546, USA; 2 Department of Toxicology and Cancer Biology, University of Kentucky, Lexington, KY 40536, USA; 3 College of Pharmacy, University of Kentucky, 789 S. Limestone Street., Lexington, KY 40506, USA; 4 Kentucky Water Resources Research Institute, 504 Rose Street, Lexington, KY 40506, USA

**Keywords:** adenine modifications, demethylase, epigenetics, gene expression, methyltransferase, nanomaterials, nematode

## Abstract

Multigenerational and transgenerational reproductive toxicity in a model nematode *Caenorhabditis elegans* has been shown previously after exposure to silver nanoparticles (Ag-NPs) and silver ions (AgNO_3_). However, there is a limited understanding on the transfer mechanism of the increased reproductive sensitivity to subsequent generations. This study examines changes in DNA methylation at epigenetic mark N6-methyl-2′-deoxyadenosine (6mdA) after multigenerational exposure of *C. elegans* to pristine and transformed-via-sulfidation Ag-NPs and AgNO_3_. Levels of 6mdA were measured as 6mdA/dA ratios prior to *C. elegans* exposure (F_0_) after two generations of exposure (F_2_) and two generations of rescue (F_4_) using high-performance liquid chromatography with tandem mass spectrometry (LC-MS/MS). Although both AgNO_3_ and Ag-NPs induced multigenerational reproductive toxicity, only AgNO_3_ exposure caused a significant increase in global 6mdA levels after exposures (F_2_). However, after two generations of rescue (F_4_), the 6mdA levels in AgNO_3_ treatment returned to F_0_ levels, suggesting other epigenetic modifications may be also involved. No significant changes in global DNA methylation levels were observed after exposure to pristine and sulfidized sAg-NPs. This study demonstrates the involvement of an epigenetic mark in AgNO_3_ reproductive toxicity and suggests that AgNO_3_ and Ag-NPs may have different toxicity mechanisms.

## 1. Introduction

Silver nanoparticles (Ag-NPs) have been increasingly incorporated into consumer products due to their antimicrobial properties [1,2,3]. These NPs are being released into the environment at different life-cycle stages of the products, including their manufacturing, production, use, and disposal, thereby increasing concerns regarding the environmental implications of these nanomaterials. There are numerous studies examining the acute, chronic, multigenerational, and transgenerational toxicity and toxicity mechanisms of various nanomaterials, including Ag-NPs in different model organisms [4,5,6,7,8,9,10]. The toxicity of Ag-NPs is partially due to the release of toxic Ag^+^ ions during NP dissolution; however, it can also be particle specific [11]. To differentiate between ionic and particulate toxicity, ionic Ag^+^ control, such as AgNO_3_, has been included in the studies with Ag-NPs. Studies on the toxicity of Ag-NPs and AgNO_3_ have been extensive in terms of the effect of nanomaterials on different biological processes and when elucidating their molecular mechanisms. However, the knowledge about their impact on the epigenome, such as DNA methylation and histone modifications, is limited. Such epigenetic changes can be used as potential biomarkers of the predictive toxicity of nanomaterials [12]. 

**Figure 1:**
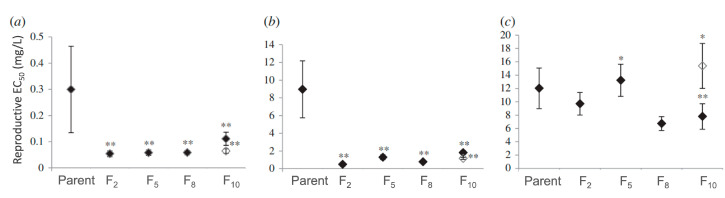
The Ag concentrations that caused a 50% decrease in reproduction (EC_50_) after *C. elegans* exposure for 2, 5, 8, and 10 generations to (**a**) AgNO_3_, (**b**) pristine Ag-NPs, and (**c**) sulfidized sAg-NPs. EC_50_ concentrations were calculated from the 3-parameter non-linear logistic regression, and the F-test was used to compare the dose–response curves between different generations and unexposed F_0_ (parents). The significant differences (*p* < 0.05 and *p* < 0.01) at each tested generation compared with F_0_ are indicated with * and ^**^, respectively. The closed diamond represents continuous exposure, and the open diamond designates the rescue treatment, where exposure was stopped after five generations. (Copyright The Royal Society (UK); Schultz et al., 2016 [17]).

Multigenerational exposures at sub-lethal concentrations, especially those including the environmentally relevant forms of Ag-NPs, are required to adequately assess environmental risks. One of the primary environmental transformations of Ag-NPs is sulfidation, which rapidly occurs through oxidation in S-rich environments, such as wastewater and soils [13,14]. Thus, organisms in the environment are often exposed to transformed sulfidized Ag-NPs (sAg-NPs) that have much lower dissolution and toxicity and distinct toxicity mechanisms when compared with pristine Ag-NPs [11,15,16]. In our previous multigenerational study with a model soil nematode, *Caenorhabditis elegans*, exposure to Ag-NPs and AgNO_3_ over multiple generations caused increased sensitivity in reproductive toxicity [17]. This heightened sensitivity was transmitted to subsequent generations even after rescue, when exposure was stopped for five more generations, demonstrating the inheritance of these toxic effects by unexposed generations. The adverse multigeneration reproductive effect of Ag is shown in Figure 1, where significantly lower EC_50_ concentrations were observed after exposure to Ag-NPs and AgNO_3_ from the F_2_ to F_10_ generations compared with the EC_50_ of the parental generation. However, for the transformed sAg-NPs, the decrease in *C. elegans* reproduction was only observed after continuous exposure at F_10_ [17]. 

To test whether such multigenerational reproductive sensitivity can be due to germline mutations, in our previous study, using whole genome DNA sequencing, mutation accumulation was compared in unexposed F_0_ and after continuous exposures in F_10_. An increased pattern in the total number of variants in all Ag treatments with significant increase in transversions for sAg-NPs was detected [18]. Thus, those results suggest that genotoxicity alone could not explain the multigenerational reproductive toxicity; therefore, epigenetic mechanisms should be investigated. In another study, we examined whether epigenetic changes at histone methylation occurred in response to Ag exposure. In that study, an increase in histone methylation at the H3K4me2 mark was observed for pristine Ag-NPs in F_3_, which also persisted in the rescue treatment in F_6_, after exposure was ceased for the subsequent three generations [19]. This indicated that epigenetic changes are involved in the observed multigenerational toxicity. 

A study with a *C. elegans* mutant strain, which lacks histone 3 lysine 4 dimethyl (H3K4me2) demethylase (*spr-5*) activity, demonstrated interplay between changes in histone methylation and DNA methylation with effects on *C. elegans* transgenerational sterility [20,21]. *Caenorhabditis elegans* mostly lacks methylation at Cytosine (5mC), and it was recently shown that DNA methylation in *C. elegans* occurs on the sixth position of the adenine ring to form N6-methyl-2′-deoxyadenosine (6mdA). In addition to the transgenerational increase in global H3K4me2 levels, the *spr-5* mutants were also shown to accumulate 6mdA in a transgenerationally inherited manner [20]. In the same study, a potential DNA methyltransferase, DNA N6-adenine methyltransferase (*damt-1*), and a DNA N6-methyl adenine demethylase (*nmad-1*) were identified. Rescue from both through the RNA interference knockdown of the methyltransferases or overexpression of the demethylases increased global levels of H3K4me2 and 6mdA and ameliorated the transgenerational sterility observed in these mutants [20,21]. These results imply a possibility of crosstalk between histone and DNA methylation in the *C. elegans* transgenerational sterility. 

The interplay between histone modifications, including histone acetylation and histone methylation, and DNA methylation have been shown to play a role in regulating gene expression, for example in human cancer cells [22,23]. Changes in DNA methylation in mammalian cell lines after exposure to different NPs have been reported [24,25,26,27,28,29]. Exposure of mouse cell lines to Ag-NPs has caused an increase in DNA methylation and upregulation of the genes encoding DNA methyltransferases, the enzymes responsible for DNA methylation [30]. The 6mdA was previously known to occur in prokaryotes but in recent studies has also been detected in multicellular eukaryotes, including plants, invertebrates, and vertebrates [31]. The findings of these studies suggest that the 6mdA is involved in response to stress, disease, and the transgenerational transfer of non-genetic information. Higher levels of adenine methylation have been detected in mitochondrial DNA vs. gDNA in mammalian cells, and these levels increased significantly in response to stress from hypoxia [32,33].

In this study, we investigated the involvement of 6mdA as a potential mechanism by which the heightened sensitivity to toxicity, observed for *C. elegans* exposed to AgNO_3_ and Ag-NPs, is transmitted to subsequent generations. We also tested whether these changes in DNA methylation can be transmitted to unexposed offspring. Sulfidized sAg-NPs were included in this study to understand the effects of the environmental transformations of pristine Ag-NPs on DNA methylation and to determine if any changes in 6mdA levels are restricted to AgNO_3_ and Ag-NPs. We hypothesized that reproductive toxic memory may be transmitted to subsequent generations through changes in epigenetic marks, such as 6mdA, which can be inherited. To our knowledge, this is the first study to examine the epigenetic effects of NP exposure on adenine DNA methylation after multigenerational exposure.

## 2. Results

### 2.1. DNA Methylation Levels

**Figure 2:**
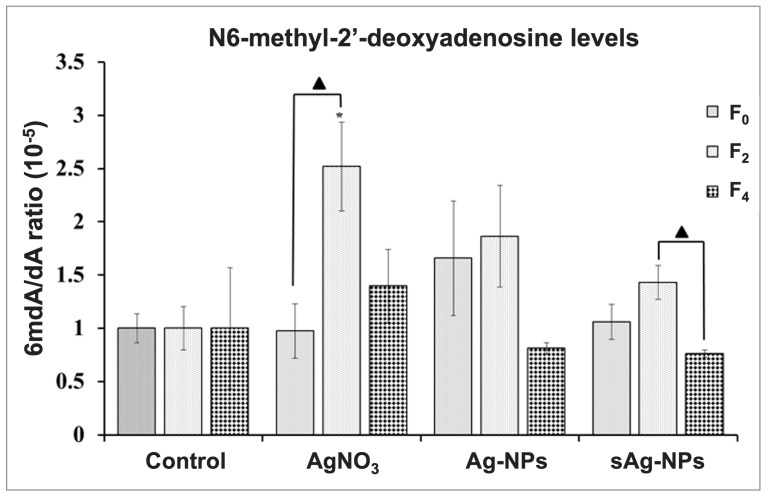
Global 6mdA levels after continuous exposure to AgNO_3_, pristine silver nanoparticles (Ag-NPs), and sulfidized Ag-NPs (sAg-NPs) assessed by HPLC-MS/MS. The exposures started at F_1_ and continued through to the F_2_ generation with rescue at unexposed F_3_ and F_4_. The Control group was not exposed to any Ag treatment for all four generations. AgNO_3_ exposure caused a significant increase in 6mdA levels. Each bar represents the mean and SEM of three biological replicates. * *p* < 0.05 when compared with controls. Filled triangle signifies difference between generations of the same treatment.

The LC-MS/MS chromatograms for standards of dA as well as 1mdA and 6mdA are shown in SI (Figure S1 and Figure S2, respectively). The retention times in mins for dA, 1mdA, and 6mdA were approximately 2.7, 1.5, and 5.7, respectively. We identified another peak in all samples that corresponded to the mass spectrometry analysis of methyl-2′-deoxyadenosine but had a lower retention time of 5.25 min compared with the 5.7 min for 6mdA (SI Figures S3–S6). The 1mdA was included to determine if this additional peak was 1mdA. However, this extra peak did not correspond to 1mdA. We were unable to acquire any other methyl-2′-deoxyadenosine standard and focused on the 6mdA peaks. Representative HPLC-MS/MS chromatograms of controls, AgNO_3_, Ag-NP, and sAg-NP-treatments for the F_0_, F_2_, and F_4_ generations are shown in Figures S3–S6, respectively. We did not detect 1mdA in any of our treatment groups (Figure S5), suggesting that the 1mdA is not involved in the reproductive toxicity induced by the Ag treatments. The global 6mdA/dA ratios for controls, AgNO_3_, pristine Ag-NPs, and sAg-NPs exposed nematodes, as determined using the standard curves, are shown in Figure 2. Significantly higher levels of global 6mdA after two generations of exposure to AgNO_3_ were detected at F_2_ (*p* = 0.035). We did not detect any significant increase in global 6mdA levels after two generations of exposure to pristine Ag-NPs (Figure 2). 

However, the global 6mdA levels of the F_0_ unexposed samples in Ag-NP treatment were higher than Control levels. Interestingly, the global 6mdA level after two generations of rescue from Ag-NP exposure (F4) returned to Control levels and was lower than the F_0_ and F_2_ levels. 

For the nematodes exposed to sAg-NPs, there were no significant changes in the levels of 6mdA after two generations of exposures (*p* = 0.179), although there was a slight increase (Figure 2). Unlike the AgNO_3_ and Ag-NP treatments, which caused an increase in sensitivity in terms of reproductive toxicity in as early as the F2 generation, sAg-NPs only did so after ten generations of exposure and at a lesser degree [17]. Therefore, we did not expect to see increased levels of 6mdA at the F_2_ generation for sAg-NP exposure. Despite no significant increases during exposure for sAg-NPs, rescue from exposure caused a significant decrease in the levels of 6mdA at F_4_ compared with the F_2_ levels (*p* = 0.015). The significant decrease may be a result of the small but not significant increase that was observed after exposures at F_2_. Despite this, after the rescue, the 6mdA levels in F_4_ were not significantly different from the F_0_ levels. Interestingly, there was also a decrease in global 6mdA levels after rescue from exposure to Ag-NPs, though it was not significant (Figure 2). This suggests that although no significant changes in 6mdA have been observed after exposure to Ag-NPs and sAg-NPs, the removal of these treatments after exposure may in itself have an effect on the nematodes. 

### 2.2. Expression of damt-1 and nmad-1

**Figure 3:**
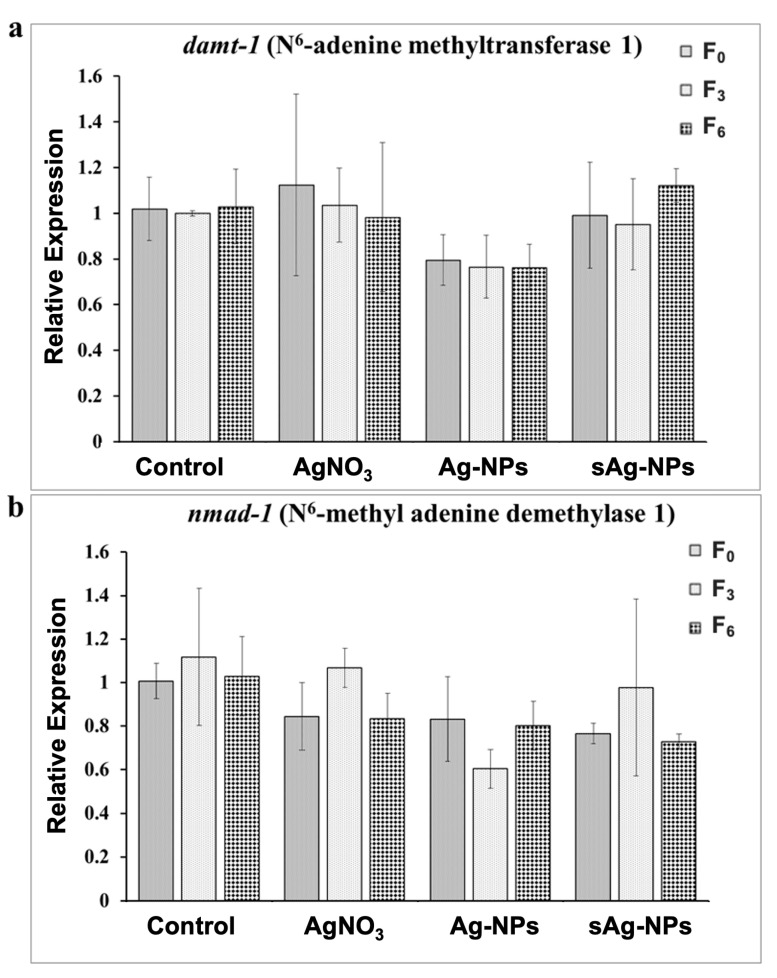
N6-adenine methyltransferase 1 (*damt-1*) (**a**) and N6-methyl adenine demethylase 1 (*nmad-1*) (**b**) gene expression levels at unexposed F_0_ after three generations of exposure at F_3_ to AgNO_3_, pristine Ag-NPs (Ag-NPs), and sulfidized Ag-NPs (sAg-NPs), and after three generations of rescue at F_6_. For the Control group, the nematodes were propagated in SSPW without Ag exposure for all six generations. Each bar represents the mean and SEM of three biological replicates.

No significant differences were observed in the mRNA levels of the *damt-1* (Figure 3a) or the *nmad-1* (Figure 3b) for any of the treatments after exposure or in the rescue treatment.

## 3. Discussion

Our results demonstrate that the exposure of *C. elegans* to AgNO_3_ caused a significant increase in the global levels of 6mdA, while the pristine Ag-NPs and sAg-NPs did not result in any significant changes in the 6mdA levels after exposure. The data from the present study correlate with our previous results, where exposure to AgNO_3_ increased multigenerational reproductive toxicity [17]. However, despite previously observing heightened sensitivity also for pristine Ag-NPs [17], as well as changes in histone methylation after Ag-NP exposure [19], we did not obtain evidence that 6mdA is involved in the response to Ag-NPs or sAg-NPs. Thus, our results indicate that the mechanisms of toxicity of AgNO_3_ might be different from those of Ag-NPs. 

The correlation of 6mdA levels with transgenerational sterility was observed by Greer et al. [21] in the *C. elegans spr-5* mutant strain. Given that an increase in 6mdA levels occurs along with an increase in multigenerational reproductive sensitivity after exposure to AgNO_3_, this result implicates the involvement of global 6mdA levels in the reproductive toxicity caused by the AgNO_3_ treatment. The increased global levels of 6mdA detected after exposure to AgNO_3_ in F_2_ generation were not inherited by unexposed generations. After two generations of rescue from AgNO_3_, the levels of 6mdA decreased to almost F_0_ levels. However, it remained slightly higher than F_0_ levels, albeit not significantly. This suggests that the nematodes do recover from the higher global levels of 6mdA observed at F_2_ after exposures but do not fully return to the Control levels. The study by Schultz et al. [17] showed that after five generations of exposure, a heritable increase in sensitivity to reproductive toxicity was observed in subsequent unexposed generations for up to five generations for AgNO_3_ and Ag-NPs. In this study, we exposed only for two generations as the previously observed increase in sensitivity was observed as early as the F2 generation. It remains to be seen if the duration of exposure determines if and how early subsequent generations may recover to normal levels of global 6mdA.

The difference in the mechanisms of transgenerational toxicity for Ag-NPs and AgNO_3_ is also supported by our previous results on histone methylation changes [19]. In that study, we demonstrated changes in histone methylation in all treatments, but the pattern and direction of such changes differed between Ag^+^ ions and pristine Ag-NPs as well as between pristine and sulfidized sAg-NPs. When comparing epigenetic changes in histone methylation and DNA methylation, it is clear that we did not observe an interplay between changes in DNA methylation and histone methylation at the specific histone methylation marks analyzed previously. Changes in 6mdA levels may have dire consequences on gene expression due to 6mdA causing site-specific RNA polymerase II transcriptional pausing [34]. Despite observing no significant changes in the global 6mdA levels after exposure to pristine Ag-NPs and sAg-NPs, it is important to note that significant changes in 6mdA levels at specific loci may not be discernable by assessing only global methylation levels. Starnes et al. [16] showed that these different Ag treatments had imparted distinct transcriptomic profiles in a sub-chronic exposure study, suggesting that changes in epigenetic modifications, which can affect gene transcription, may occur in a loci-specific manner. The changes in both methylation and demethylation can occur at different sites of DNA. Thus, future studies should examine targeted changes in DNA methylation by performing a genome-wide DNA methylation profile using Methylated DNA Immunoprecipitation and Sequencing (MeDIP-Seq). This may also reveal specific genes affected by 6mdA in the different Ag treatments. 

In Ag-NP treatment, it is important to note that the higher levels of global 6mdA at the F_0_ generation may have masked any significant increase in 6mdA levels that may have been induced by the Ag-NP treatment. However, we failed to obtain any conclusive evidence that Ag-NPs induced significant changes in global 6mdA levels in this study. Future studies on DNA methylation should focus on loci-specific changes, as changes in DNA methylation patterns may not be sufficient to detect from global DNA methylation analysis. 

Considering the significant increase in 6mdA/dA ratios observed after exposure to AgNO_3_, we expected upregulation of the methyltransferase *damt-1* and/or a downregulation of demethylase *nmad-1*. However, we have not observed any significant changes in the expression of these genes. Up to three major DNA methyltransferases (DNMT-1, DNMT-3a, and DNMT-3b) can catalyze the methylation of cytosine to 5-methylcytosine [30]. While DNMT-1 maintains established methylation patterns, DNMT-3a and DNMT-3b are responsible for de novo DNA methylation [30,35,36]. DNA N6-adenine methyltransferase 1 (*damt-1*) is an orthologue of human methyltransferase-like protein 4 (METTL-4), which is involved in RNA N6-adenosine methylation [21]. METTL-4 has mammalian homologs, such as METTL-3 and METTL-14, which have been shown to have higher methylation activity [37]. It is therefore possible that other DNA N6-adenine methyltransferases are yet to be identified that may be involved in de novo DNA methylation under stress conditions. It should also be noted that gene expression levels were measured after three generations of exposure and three generations of rescue, whereas 6mdA levels were measured after two generations of exposure and rescue. Thus, the gene expression levels may change only transiently at the beginning of exposures, which might also explain why the increase in the DNA methylation level in AgNO_3_ treatment was not inherited. Time gene expression analysis during exposures would be warranted to determine it. 

This study was designed to investigate the potential changes in global DNA methylation (6mdA) levels, which may explain at least in part, the multi- and transgenerational increase in sensitivity to reproductive toxicity observed after exposures of *C. elegans* to AgNO_3_ and Ag-NPs. This is one of the first studies to show that DNA adenine methylation is involved in stress induced by metal (Ag) toxicity in *C. elegans*. Overall, our results demonstrate that exposure to AgNO_3_, but not pristine or transformed Ag-NPs, causes a significant increase in the global levels of 6mdA. This also suggests that for AgNO_3_, after multigenerational exposure, there is an association between changes in epigenetic DNA adenine methylation marks and enhanced reproductive toxicity. For Ag-NPs, however, based on our previous work and this study, epigenetic changes were documented at global histone methylation levels. Taken together, this highlights the differences in the epigenetic mechanisms of multigenerational reproductive toxicity between Ag^+^ ions and Ag-NPs. 

## 4. Materials and Methods

### 4.1. Silver Nanoparticle Synthesis and Characterization

Polyvinylpyrrolidone (PVP)-coated Ag-NPs were synthesized as previously described [38]. The same batch of Ag-NPs used by Starnes et al. [11] and Schultz et al. [17] was used in this study. The stability of Ag-NPs was confirmed by performing dynamic light scattering (DLS) analysis, which yielded a nearly identical primary particle size, and the dissolution rate was similar to what has been observed in the previous studies. Sulfidation was carried out fresh by combining Ag-NPs with Na_2_S at a 2:1 molar ratio of S to Ag. The mixture was incubated at room temperature for 4 h open to the atmosphere. The tube was capped and sealed and incubated at room temperature for an additional seven days. The sAg-NPs were separated from the reaction solution and washed thrice with 18.2 MΩ deionized water. Complete sulfidation was confirmed by powder X-ray diffraction (X’Pert Pro, Malvern PANalytical, Malvern, UK).

Characterization of Ag-NPs is described in our previous studies. The transmission electron microscopy (TEM) primary particle sizes were reported to be 58.3 ± 12.9 nm for PVP-coated Ag-NPs and 64.5 ± 19.4 nm for sAg-NPs [17]. Upon addition of Ag-NPs and sAg-NPs into the simulated soil pore water (SSPW) used for exposures, the volume weighted sizes were determined by dynamic light scattering to be 66.26 ± 34.33 nm and 60.73 ± 20.67 nm, respectively. The zeta potentials of Ag-NPs and sAg-NPs in the SSPW were −5.3 mV and −15.7 mV, respectively. From our previous multigenerational study, which used the same NPs in the same media (SSPW), the dissolution after 96 h determined via ultrafiltration was 1.5 ± 0.1% for Ag-NPs and 0.023 ± 0.002% for sAg-NPs [17].

### 4.2. Nematode Exposures

**Figure 4:**
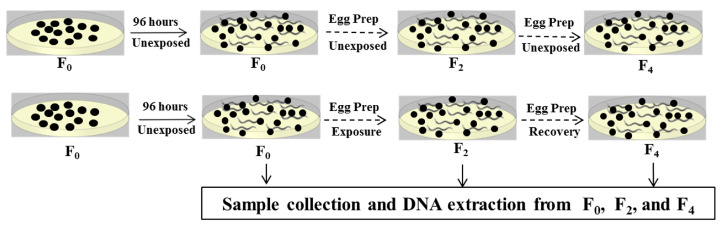
Experimental design to examine changes in the global level of DNA adenine methylation in Controls and after exposure of *C. elegans* to AgNO_3_, pristine silver nanoparticles (Ag-NPs) and sulfidized Ag-NPs (sAg-NPs). DNA was extracted from F_0_, F_2_ and F_4_. The dashed arrow indicate that propagation of the lineage continued through the next generation.

*Caenorhabditis elegans* (N2) was acquired from the Caenorhabditis Genetics Center (University of Minnesota, USA). In this study, exposures were carried out on a population of nematodes, which were propagated for multiple generations as illustrated in Figure 4. DNA methylation (6mdA) levels were analyzed prior to exposure (F0), after two generations of exposure (F2), and after two generations of rescue from exposure (F4) to determine the influence of the different forms of Ag exposures on 6mdA levels (Figure 4). Exposures were carried out in simulated soil pore water (SSPW) to mimic natural soil solution condition [17,39]. First, exposure to pristine Ag-NPs was carried out in a separate experiment followed by exposures to AgNO_3_ and sAg-NPs in the same experiment. There was no Ag exposure at any generation in the Control group, which was also started from F_0_ and was propagated in SSPW for four generations with DNA samples extracted at F_0_, F_2_ and F_4_ generations. Unexposed F_0_ was included within each treatment.

Age synchronization using NaClO/NaOH solution was performed following the protocol [40]. Briefly, the nematodes were washed off the 6 cm K-agar plates into 15 mL tubes using K-medium and centrifuged for 1 min. Then, the supernatant was taken out and the NaClO/NaOH solution was added. The gravid nematodes were dissolved by shaking the tubes for 6 min while the eggs were more resistant to this procedure. After washing twice with K-medium, the eggs were placed in 10 cm SSPW agar plates to start the unexposed F_0_ populations of three replicates per treatment group (Control, AgNO_3_, pristine Ag-NPs, and sAg-NPs). Nematodes were fed dam-dcm-*E. coli* strain (NEB C2925), which lacks methyltransferases that carry out adenine and cytosine methylation. *E. coli* suspended in 6 mL of SSPW at an optical density (540 nm) of 0.35 was added to each plate as a food source and incubated at 20 °C. After 96 h, nematodes were washed off the plate into 15 mL centrifuge tubes using SSPW and split in half. Half of the nematodes were saved for DNA extraction and half used for age synchronization to obtain eggs for the next generation (F_1_). It is important to clarify that nematodes in the liquid media delay their development, and it takes 96 h from the egg to reach gravid adult stage in SSPW. Exposures were started at the F_1_ generation with the SSPW/dam-dcm-*E. coli* food source dosed with equitoxic concentrations (EC_30_ for reproduction as determined from dose–response experiments [17] and reconfirmed in this experiment) of AgNO_3_ (0.07 mg/L), Ag-NPs (1.5 mg/L), and sAg-NPs (6 mg/L). We selected a sub-lethal concentration, where reproductive toxicity was observed. Silver concentration above EC_30_ would have resulted in mortality and prevented propagation of *C. elegans* over multiple generations. Our previous epigenetic multigenerational study on changes in histone methylation was conducted with the same Ag-NPs and also used EC_30_ for exposures [19]. Having the same equitoxic Ag concentrations in both studies allows us to make comparisons between the epigenetic changes and their interrelationship (or lack thereof) observed between different type of epigenetic changes in response to Ag exposure. The SSPW/OP50 solution without NPs or AgNO_3_ was used for controls throughout the experiment. Fresh OP50 in SSPW was added after each age synchronization step in every treatment, when starting a subsequent generation. 

#### 4.2.1. Exposure Scenario for Measuring DNA Adenine Methylation Levels

Exposures were carried out for two generations (F_1_ and F_2_). Each generation was exposed for 96 h, after which age synchronization was performed to obtain eggs for each subsequent generation. After the last exposure (F_2_), nematodes were washed off the plate with SSPW and half of the nematodes were saved for DNA extraction. The other half was used for age synchronization to obtain eggs to start the F_3_ generation, at which point rescue from exposure was started by feeding nematodes with SSPW/dam-dcm-*E. coli* solution not spiked with any of the Ag treatments. The rescue was carried out for two generations (F_3_ and F_4_), at which point the experiment was terminated. The F_4_ populations were washed off the plate with SSPW and used for DNA extraction.

#### 4.2.2. Exposure Scenario for Measuring Changes in Gene Expression

Given that it is extremely difficult to collect sufficient amounts of DNA and RNA from the same generation and to have sufficient population for propagation, RNA samples for measuring changes in gene expression were used from a separate experiment. Here, we were able to maintain continuous exposure at EC_30_ and successfully propagate nematodes for an extra generation (F_1_ to F_3_). After exposures were stopped at F_3_, half of the nematodes were used for RNA extractions, with the other half used for age synchronization to obtain the F_4_ generation. The rescue started at unexposed F_4_ and continued for three more generations (F_4_ to F_6_). The nematodes from unexposed F_0_, exposed F_3_, and after rescue at F_6_ were used for RNA extractions. The duration of exposure and rescue was the same length. We also included a separate Control group for this experiment, starting from F_0_, propagating nematodes for six generations, and extracting RNA at F_0_, F_3_, and F_6_ generations.

### 4.3. DNA Extractions

Nematodes were washed thrice using DI water with gentle centrifugation at 800 rpm for 1 min to get rid of residual bacteria. After the final wash, the nematodes were used for DNA extraction using Qiagen DNeasy Blood & Tissue Kit (QIAGEN, Hilden, Germany) with modifications. Six free-thaw cycles were performed, and 20 µL of proteinase K was added. The samples were incubated for 3 h at 56 °C and briefly vortexed every 15 min for 15 s. Proteinase K was deactivated at 90 °C for 10 min. DNA was then treated with RNase A/T1 mix (Thermo Scientific, Waltham, MA, USA) at a 1:20 dilution and RNaseH (NEB) at a 1:50 dilution for 1 h at 37 °C. The samples were transferred to the Qiagen columns and DNA was eluted with 150 µL of TE (10 mM Tris-HCl, 1 mM EDTA, pH 7.5) and stored at -20 °C.

### 4.4. Quantification of 6mdA in Genomic DNA by LC-MS/MS Analysis

The method for 6mdA quantification was adapted from Greer et al. [21]. Extracted genomic DNA was concentrated and re-suspended in nuclease-free H_2_O water using DNA Clean & Concentrator-5 Kit (Zymo Research, Irvine, CA, USA) as described by the manufacturer. DNA samples were eluted using 45 µL of DI water. DNA concentrations were measured using the DNA Quantitation Kit Fluorescence Assay (DNAQF, Sigma-Aldrich, St. Louis, MO, USA) according to the manufacturer’s recommendations. Fluorescence was measured using a DyNA Quant 200 fluorometer (Hoefer Pharmacia Biotech Inc., San Francisco, CA, USA). 

*Caenorhabditis elegans* genomic DNA (1–9 µg) in 40 µL of nuclease-free H2O was digested to free nucleosides using 5 IU of DNA degradase plus (Zymo Research) in 50 µL reactions for 4 h at 37 °C. For calibrations standards, N6-methyl-2′-deoxyadenosine (6mdA) and 2′-deoxyadenosine (dA) (Thermo Scientific) were digested using 5 IU of DNA degradase plus in 50 µL reactions for 4 h at 37 °C. After digestion of the standards and samples, the volume was brought up to 80 µL with DI water. The samples were centrifuged at 14,000 rpm for 15 min, after which 70 µL of the supernatant was transferred into fresh vials. A total of 20 µL of each sample was analyzed by reverse-phase high-performance liquid chromatography with tandem mass spectrometry (HPLC-MS/MS).

The nucleosides were separated by reverse-phase HPLC using a Varian ProStar 210 HPLC system with a phenomenex kinetex C18 reversed-phase column (100 × 2.1 mm, 2.6 µm). Mobile phase A was water with 0.1% (*v*/*v*) formic acid and mobile phase B was methanol with 0.1% (*v*/*v*) formic acid. The gradient used was 8 min from 98% phase A and 2% phase B to 5% phase A and 95% phase B for 4 min, followed by 10 min post-run with 98% phase A and 2% phase B. Online mass spectrometry detection was performed using a Varian 1200 L triple quadrupole mass spectrometer in positive electrospray ionization mode. Quantification was accomplished in multiple reaction monitoring (MRM) by monitoring the transitions of 266.0–150.0 (6mdA) and 252.0–136.0 (dA). Quantification of the ratio of 6mdA/dA was performed using calibration curves obtained using nucleoside standards acquired from Alfa Aesar (Haverhill, MA, USA). More detailed information about the calibration standards with their respective chromatograms is presented in SI.

### 4.5. Quantitative Real-Time Polymerase Chain Reaction Analysis

Expression levels of DNA N6-adenine methyltransferase 1 (*damt-1*) and N6-methyl adenine demethylase 1 (*nmad-1*) were investigated. RNA was extracted using Qiagen Kit from *C. elegans* prior to exposure (F_0_), after three generations of exposure (F_3_), and after three generations of rescue from exposure (F_6_) to AgNO_3_, Ag-NPs, and sAg-NPs. cDNA synthesis was carried out with 500 ng of total RNA by using RevertAid First Strand cDNA Synthesis Kit (Thermo Scientific, Waltham, MA, USA). qRT-PCR reactions were performed in 10 µL volume with TaqMan fast advanced master mix, TaqMan gene expression assays for each gene, and cDNA diluted 1:19. The optimal cDNA dilution factor was determined based on the dilution amplification curves from the efficiency tests. StepOne Plus system (Applied Biosystems, Waltham, MA, USA) was used for all amplifications with a program of 10 min at 95 °C, followed by 40 cycles of 10 s at 95 °C and 30 s at 60 °C. All treatments for each gene were run in triplicates. Negative controls and minus reverse transcriptase (-RT) negative controls were run for every gene/sample to check for DNA contamination.

The TaqMan primer/probe assay IDs and their amplification efficiencies are shown in SI (Table S1). Y45F10D.4 gene, a putative iron-sulfur cluster assembly enzyme, was used as the reference gene due to its highly stable expression levels [41]. Its expression levels were also stable among all Ag treatments used with 0.5–1 Ct difference (SI Figure S7). The data were exported into GenEx^TM^ 6.0 software (MultiD), and after normalization to the reference gene, the expression levels of the target genes relative to controls were calculated following the Pfaffl method [42].

### 4.6. Data Analysis

SAS was used for the statistical analyses. The data are given as mean and standard errors of the mean. One-way ANOVA with Tukey’s post hoc test was used to test for the statistical significance among treatments. The differences with *p* < 0.05 were considered as statistically significant. The data for 6mdA/dA were arcsine square root transformed prior to running ANOVA.

## Data Availability

Data supporting reported results presented in the study are included in the manuscript and Supplementary Material; further inquiries can be directed to the corresponding author.

## Supplementary Materials

The supporting information can be downloaded at: https://www.mdpi.com/article/10.3390/ijms24076168/s1.
