# Supplementary material for: Global DNA Adenine Methylation in Caenorhabditis elegans after Multigenerational Exposure to Silver Nanoparticles and Silver Nitrate
Source: Int J Mol Sci. 2023 Mar 24;24(7):6168. doi: 10.3390/ijms24076168 (PMC10094302; doi:10.3390/ijms24076168)
Supplement: Supplementary file 1 [file ijms-24-06168-s001.zip › ijms-2240346-supplementary.pdf]

Supplementary Information

Global DNA adenine methylation in *Caenorhabditis elegans* after multigenerational exposure to silver nanoparticles and silver nitrate

Anye Wamucho [1,2,3], Jason Unrine [1,4], John May [1] and Olga Tsyusko [1,*]

[1] Department of Plant and Soil Sciences, University of Kentucky, Lexington, KY 40546, USA; a.wamucho@uky.edu (A.W.); jason.unrine@uky.edu (J.U.); john.may@uky.edu (J.M.)
[2] Department of Toxicology and Cancer Biology, University of Kentucky, Lexington, KY 40536, USA
[3] College of Pharmacy, 789 S Limestone Street, University of Kentucky, Lexington, KY 40506, USA
[4] Kentucky Water Resources Research Institute, 504 Rose Street, Lexington, KY 40506, USA
* Correspondence: olga.tsyusko@uky.edu

LC-MS/MS Chromatograms.

The chromatograms of the 2′ Deoxyadenosine (dA) standard are shown in **Figure S1** and the chromatogram of the N^1-methyl-2′- deoxyadenosine (1mdA) and N^6-methyl-2′- deoxyadenosine (6mdA) standards are shown in **Figure S2.** Representative chromatograms of one replicate for all generations for controls, $AgNO_3$, pristine Ag-NPs, and sAg-NPs are shown in **Figures S3-S6** respectively.

Standard concentrations were selected based on *Caenorhabditis elegans* DNA sample test runs. Standards were ran through the same digestion process as the *C. elegans* DNA samples before subjected to HPLC-MS/MS and used to generate 1mdA and 6mdA linear standard curves for quantification of *C. elegans* 1mdA and 6mdA levels respectively. Quantification was accomplished in multiple reaction monitoring (MRM) by monitoring the transitions 266.0–150.0.

For the samples, a second peak corresponding to the monitored 266.0 to 150.0 transition was detected with a slightly lower retention time (approximately 5.25 minutes) compared to 6mdA as seen in **Figures S3-S6**. Test samples spiked with 6mdA prior to HPLC-MS/MS runs indicated that the peak does not correspond to 6mdA (data not shown). The retention time of the peak did not also correspond to 1mdA suggesting another potential methyl-2′-deoxyadenosine base in the *C. elegans* genome, potentially methylated at another position on the adenosine ring. dA and 6mdA levels were quantified using standard curves generated from the standards ran at the same time with the samples.

Table S1 Assay IDs and efficiencies of TaqMan primer/probes used for qRT-PCR

Gene symbol	Gene name	Assay ID	Efficiency (%)
damt-1 (C18A3.1)	DNA N^6-methyl adenine methyltransferase	Ce02432992_g1	100.7
nmad-1 (F09F7.7)	DNA N^6-methyl adenine demethylase	Ce02406554_g1	97.1
Y45F10D.4	Uncharacterized	Ce02467252_g1	100

Figures

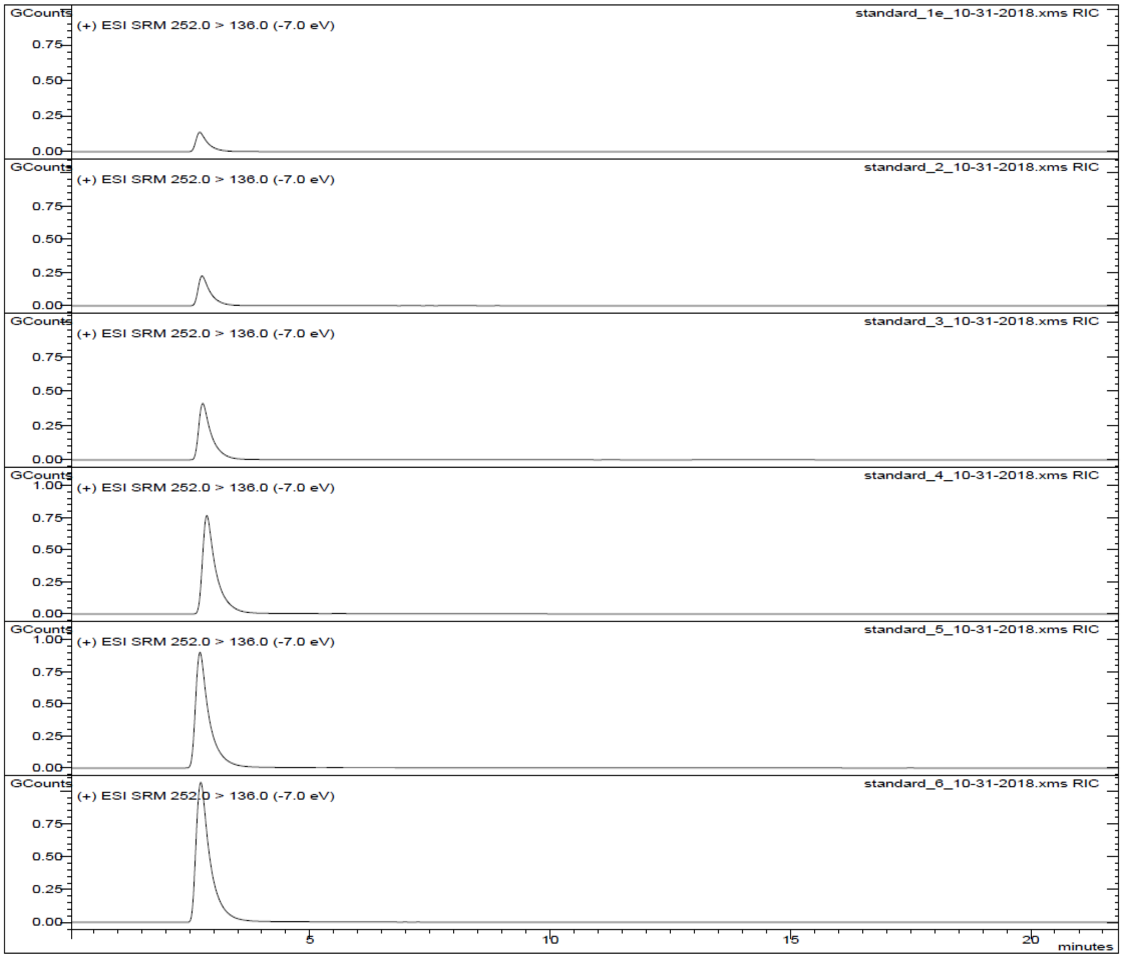

Figure S1. HPLC-MS/MS chromatograms of 2′ Deoxyadenosine (dA) standard. The concentration for dA standards was between 0.5 mg/L – 10 mg/L. dA had a retention time of 2.7 minutes.

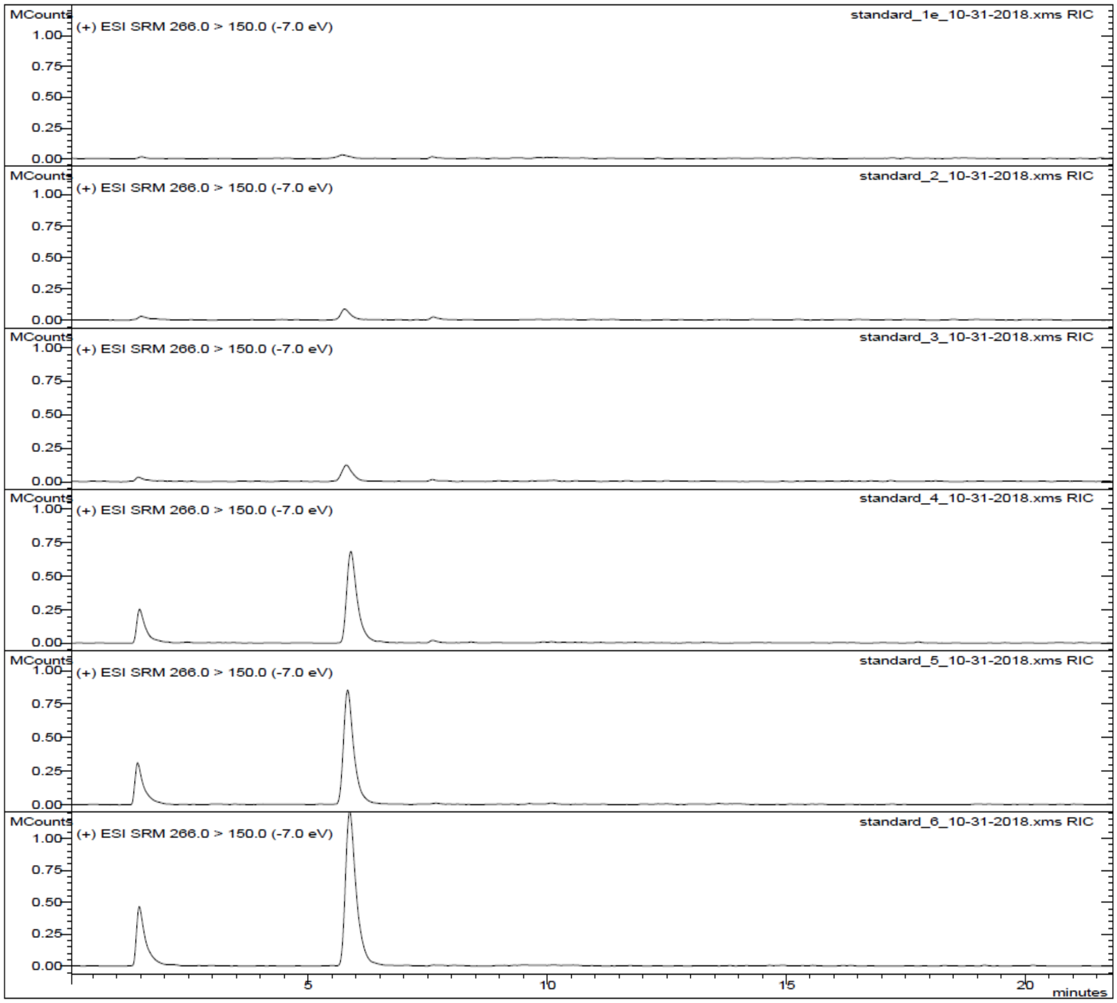

Figure S2. HPLC-MS/MS chromatograms of N[1]-methyl-2′- deoxyadenosine (1mdA) and N[6]-methyl-2′- deoxyadenosine (6mdA) standards. The concentration for both standards was between 0.025 μg/L – 1 mg/L. 1mdA had a retention time of 1.5 minutes and 6mdA had a retention time of 5.7 minutes.

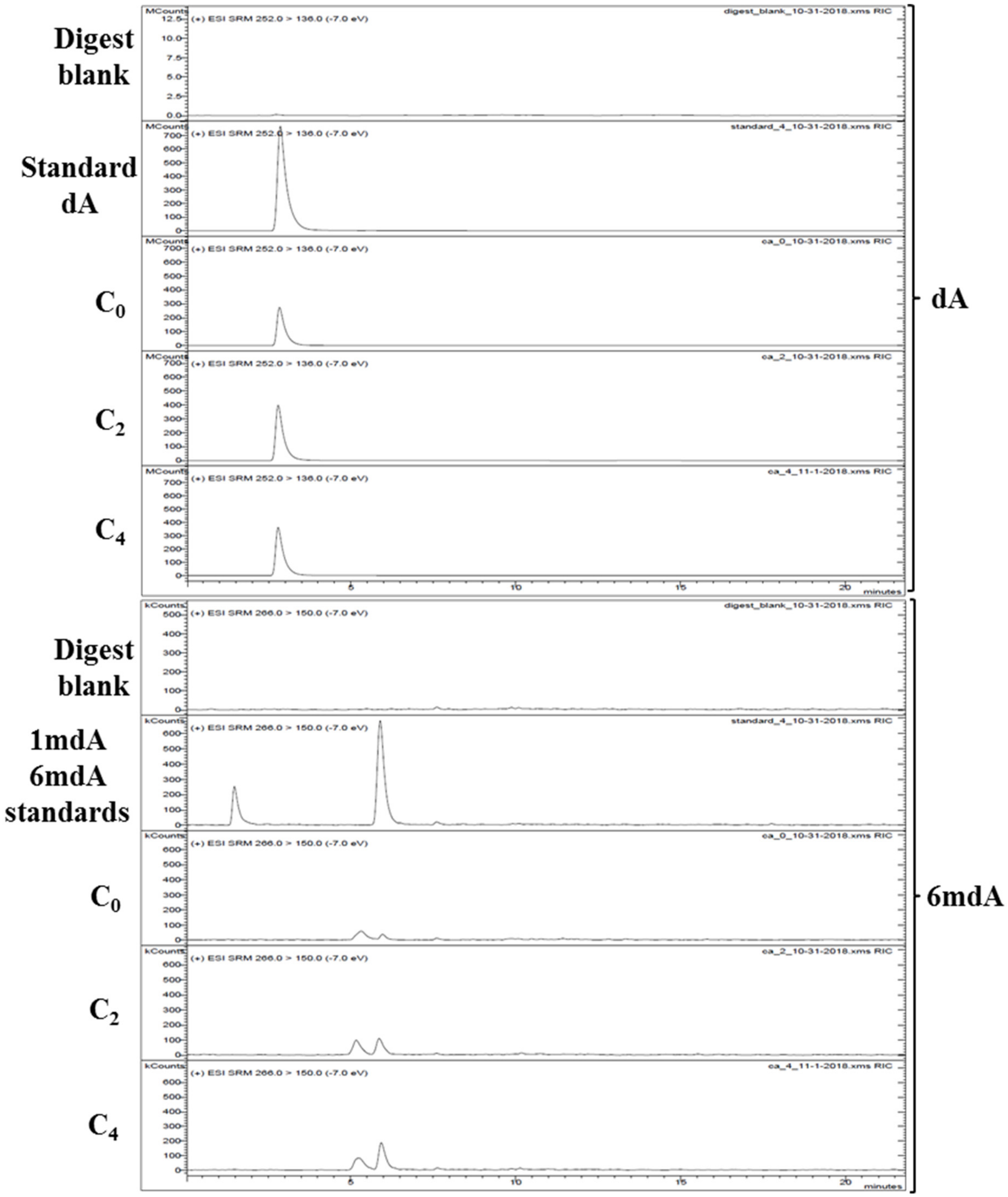

Figure S3. Representative HPLC-MS/MS chromatograms of 2′ deoxyadenosine (dA), N^1-methyl-2′- deoxyadenosine (1mdA), and N^6-methyl-2′- deoxyadenosine (6mdA) of **control** *C. elegans* DNA samples. 1mdA was not detected for any sample.

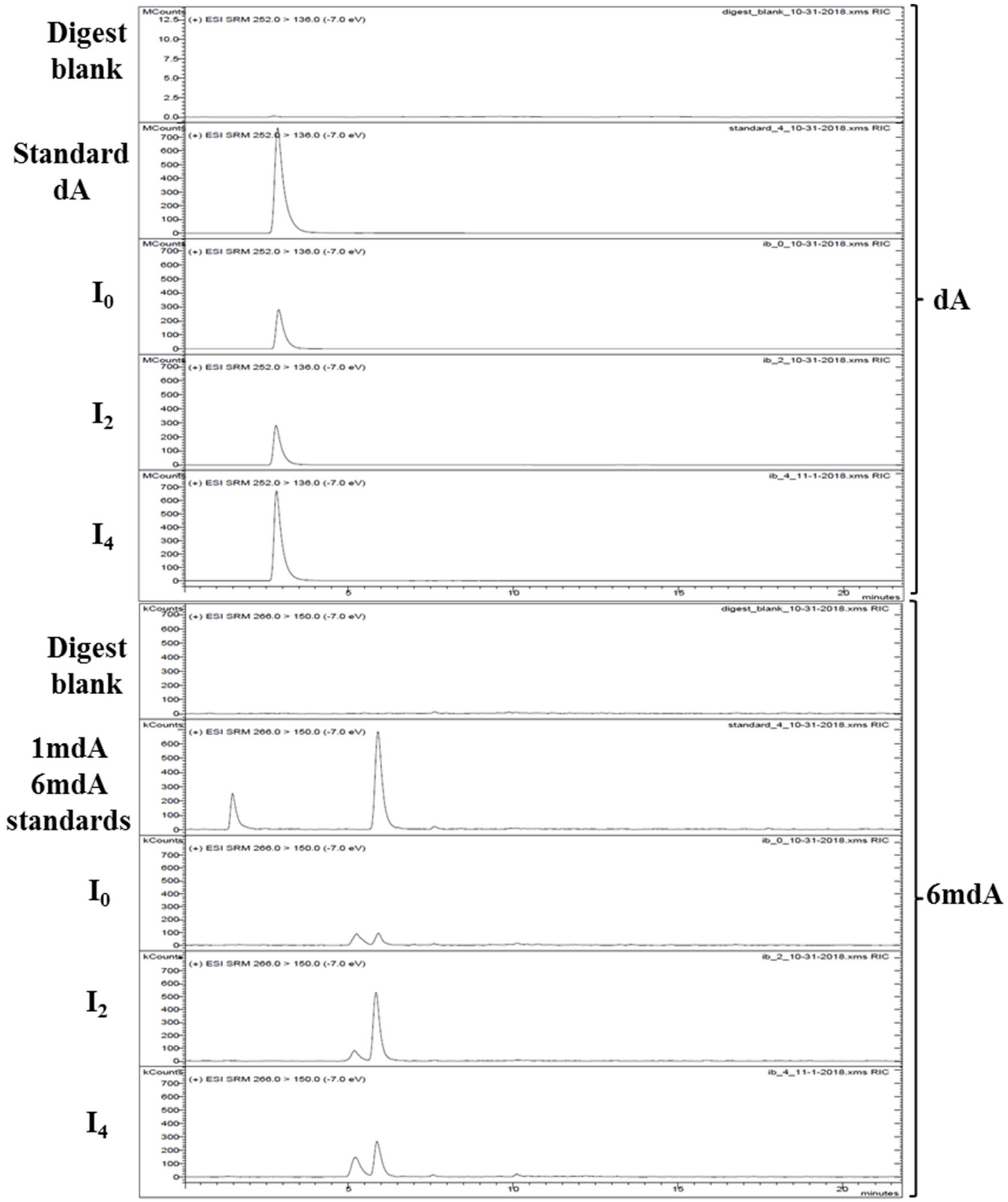

Figure S4. Representative HPLC-MS/MS chromatograms of 2′ deoxyadenosine (dA), N1-methyl-2′- deoxyadenosine (1mdA), and N6-methyl-2′- deoxyadenosine (6mdA) of **$AgNO_3$ (I) exposed** *C. elegans* DNA samples. 1mdA was not detected for any sample. 6mdA levels after two generations of exposures (F_2) was significantly higher compared to the unexposed F_0 as determined by the 6mdA/dA ratio. 6mdA levels retuned to almost F_0 levels after rescue for two generations from exposure (F_4).

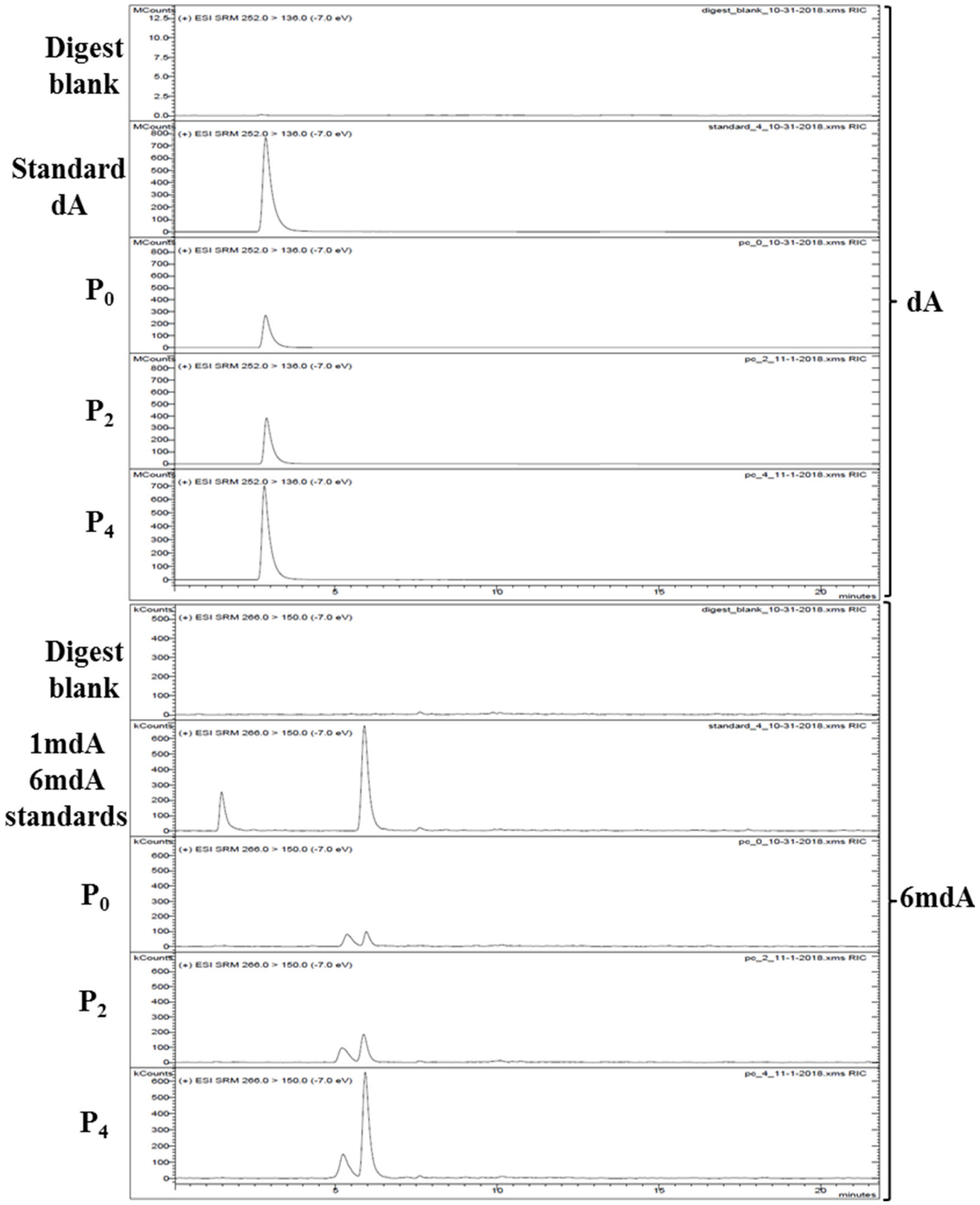

Figure S5. Representative HPLC-MS/MS chromatograms of 2′ deoxyadenosine (dA), N^1-methyl-2′- deoxyadenosine (1mdA), and N^6-methyl-2′- deoxyadenosine (6mdA) of **pristine Ag-NPs (P) exposed** *C. elegans* DNA samples. 1mdA was not detected for any sample.

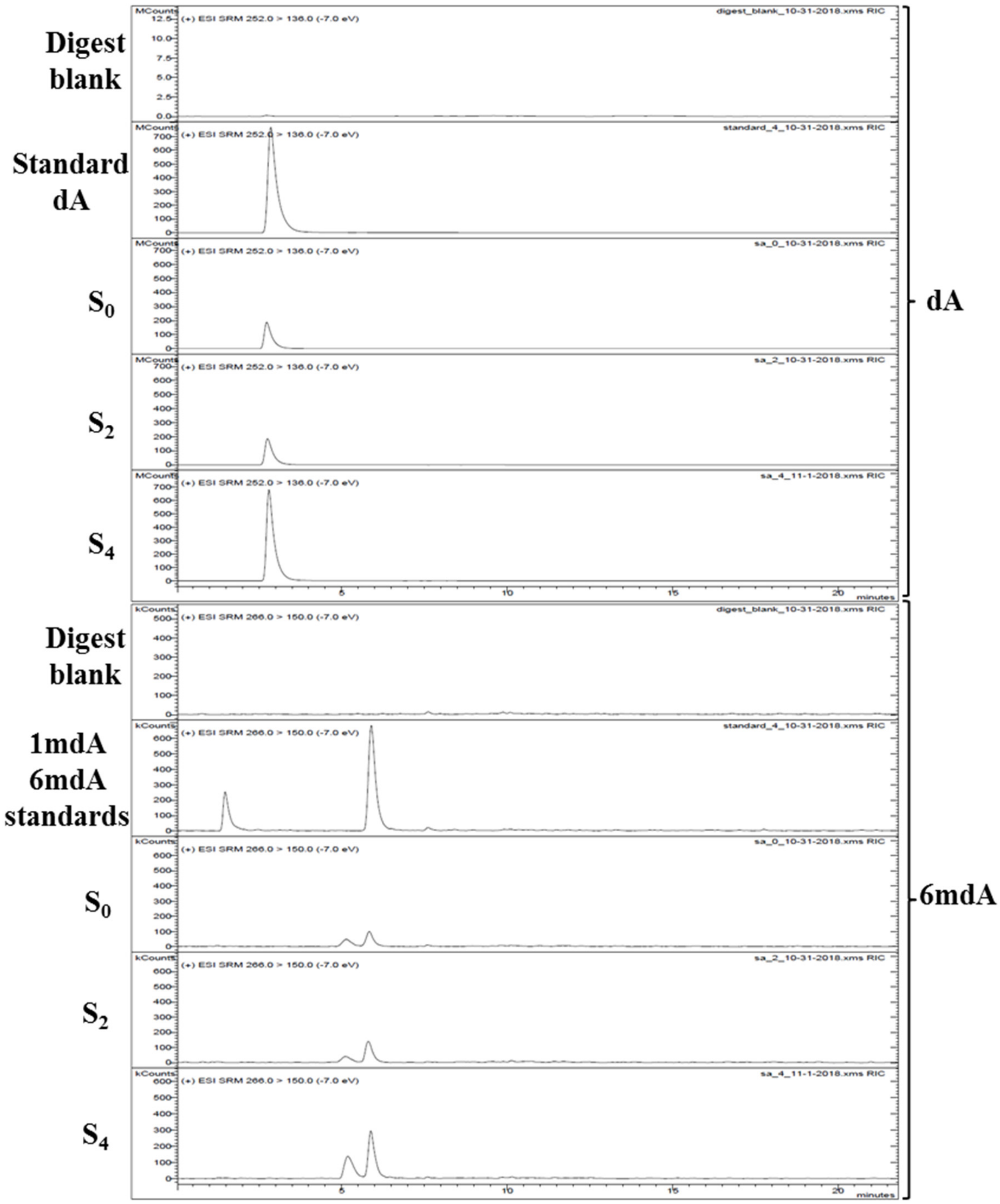

Figure S6. Representative HPLC-MS/MS chromatograms of 2′ deoxyadenosine (dA), N1-methyl-2′- deoxyadenosine (1mdA), and N6-methyl-2′- deoxyadenosine (6mdA) of **sulfidized (S) Ag-NP exposed** *C. elegans* DNA samples. 1mdA was not detected for any sample.

Stability of the Reference Gene: An equivalent amount of total RNA from *Caenorhabditis elegans* exposed to control, $AgNO_3$, pristine Ag-NPs, and sAg-NPs was converted to cDNA and the cDNA diluted up to 20-fold for qRT-PCR. The amplification curves of Y45F10D.4 gene under different treatments showed stable expression, indicating it can be used as a reference gene for relative quantification in our study.

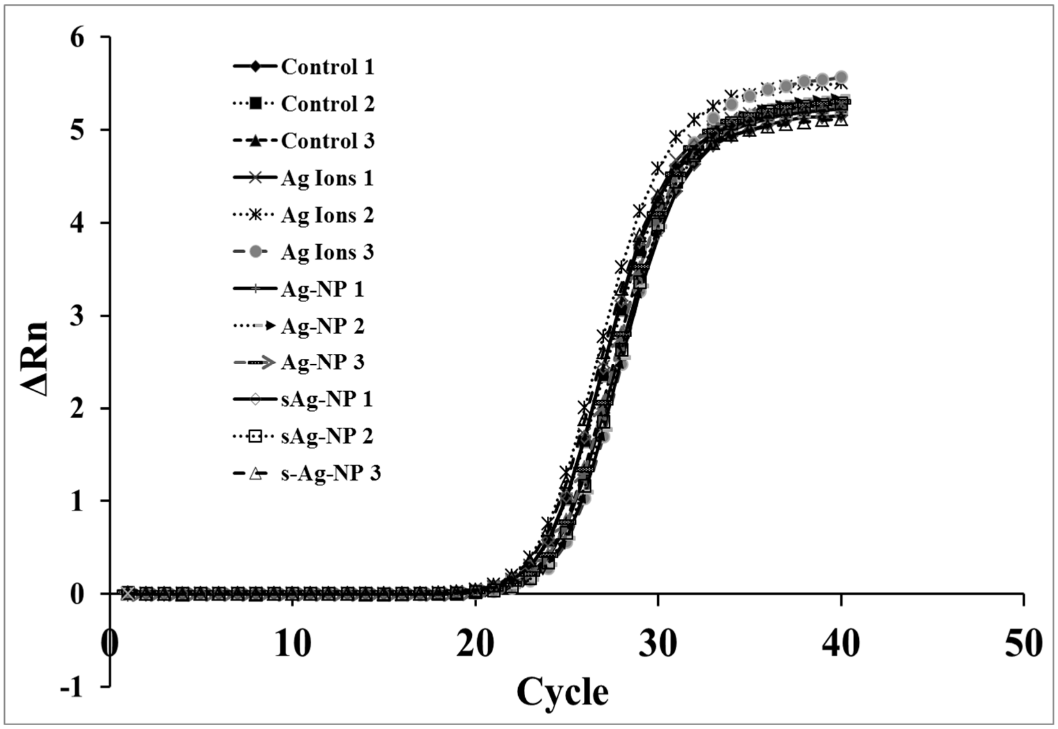

Figure S7. The quantitative real-time PCR (qRT-PCR) amplification curves of reference Y45F10D.4 gene expression in *C. elegans* from control, $AgNO_3$ (Ag^+ Ions), pristine Ag-NPs, and sulfidized Ag-NP (sAg-NPs) treatments.
